# Accelerometer‐measured physical activity, sedentary behavior, and incident neuropsychiatric diseases: a large prospective cohort study of 73411 participants

**DOI:** 10.1002/alz70856_102961

**Published:** 2025-12-25

**Authors:** Wu Jiayi, Jin‐Tai Yu

**Affiliations:** ^1^ Fudan University, Shanghai, Shanghai, China; ^2^ Huashan hospital, Fudan University, Shanghai, China

## Abstract

**Background:**

Physical activity and sedentary behavior are closely associated with neuropsychiatric diseases, while previous studies have mainly relied on self‐reported data, which have been shown to be inconsistent with objectively measured metrics.

**Method:**

This large‐scale prospective study used accelerometer data from 73,411 participants collected between 2013 and 2015, focusing on energy expenditure and time allocation.

**Result:**

A total of 73,411 participants (mean and standard deviation age: 56.08 ± 7.82 years; 40,902 (55.72%) women; 70,771 (96.40%) white) were included. Moderate to vigorous physical activity energy expenditure showed the strongest protective effect on neuropsychiatric diseases (hazard ratios: 0.60‐0.86, all FDR‐Q<0.001), while an increased proportion of sedentary time was identified as a risk factor (HRs: 1.05‐1.54, FDR‐Q<0.05). Restricted cubic spline analyses demonstrated an L‐shaped association linking physical activity and neuropsychiatric diseases and an inverted L‐shaped relationship between sedentary behavior and dementia. Linear regression models further linked physical activity and sedentary behavior to brain functions. Key brain regions related to these behaviors included the lateral occipital cortex, cuneus, pallidum, and accumbens. Proteomics and metabolic analyses identified significant involvement of ITGAV protein and high‐intensity lipoprotein. Structural equation modeling elucidated that inflammation and metabolism mediate these relationships.

**Conclusion:**

In summary, we underscored the protective role of higher physical activity energy expenditure, increased engagement in physical activity, and reduced sedentary behavior in mitigating the risk of five neuropsychiatric diseases, regardless of activity intensity. Notably, we investigated the underlying mechanisms, confirmed associations with brain functions, brain structures, and peripheral biomarkers, and highlighted the mediating role of inflammatory and metabolic markers in these associations. These findings have significant implications for assessing risk factors and developing preventive interventions for neuropsychiatric diseases.

